# Food-derived antioxidant peptides undergo oxidative transformations that may alter their function and safety

**DOI:** 10.1007/s44345-026-00063-x

**Published:** 2026-07-03

**Authors:** Matthew D. Ezema, Harthika Mylvaganam, Apollinaire Tsopmo

**Affiliations:** 1 Food Science, Department of Chemistry, Carleton University, 1125 Colonel By Drive, Ottawa, K1S 5B6 ON Canada; 2 Department of Biochemistry, Federal University, Oye-Ekiti, Ekiti State Nigeria; 3 Institute of Biochemistry, Carleton University, 1125 Colonel By Drive, Ottawa, ON Canada

**Keywords:** Oxidative modification, Lipoxidation, Glycoxidation, Toxicity assessment, Structure-activity, Cytotoxic peptide

## Abstract

Bioactive peptides used in foods and nutraceuticals are frequently exposed to oxidizing environments that reshape their structure, function, and safety profile. Beyond acting as radical scavengers, peptides can undergo extensive modification by reactive oxygen species (ROS) and by oxidized lipid and sugar products that co-occur in complex food matrices. Mechanistic studies reveal that hydroxyl radicals, peroxyl radicals, and metal‑catalyzed H_2_O_2_ systems generate peptide hydroperoxides, carbonyls, backbone fragments, and covalent dimers in a sequence‑dependent manner, particularly at proline, tryptophan, tyrosine, and histidine residues. Parallel reactions with oxidized lipids, including lipid hydroperoxides and electrophilic aldehydes such as malondialdehyde and hydroxynonenal, drive additional layers of modification through trans‑oxidation, Schiff base formation, and Michael addition, yielding diverse adducts whose structures and stabilities differ markedly from classical ROS products. Glycation and glycoxidation further contribute carbonyl‑derived cross‑links and fluorophores in sugar‑rich systems. While the biological consequences of protein oxidation are well described, the functional and toxicological implications of oxidized bioactive peptides remain largely unexplored. Emerging evidence indicates that peptide hydroperoxides, lipid‑derived aldehyde adducts, and advanced glycoxidation/lipoxidation products can alter peptide bioavailability, immunoreactivity, redox behaviour, and cellular functions in cell and animal models. This review integrates current knowledge on the chemistry, analytical detection, and structural diversity of peptide oxidation products, highlights sequence‑specific reactivity patterns, and outlines safety considerations and research priorities for peptide‑based functional ingredients.

## Introduction

Food-derived bioactive peptides have gained considerable attention as multifunctional molecules with applications across the food, nutraceutical, and pharmaceutical sectors. These short amino acid sequences, naturally encrypted within plant and animal proteins, exhibit a broad range of physiological activities, including antihypertensive, anticancer, antimicrobial, immunomodulatory, cholesterol-lowering, and antioxidant effects [1, 2]. Their natural origin, structural versatility, and compatibility with diverse delivery systems make them attractive candidates for incorporation into functional foods and health-promoting formulations [2]. Among these bioactivities, antioxidant potential has drawn particular interest due to its relevance in preventing oxidative deterioration and restoring the oxidation-reduction (redox) balance. This is because oxidative stress, an imbalance between reactive oxygen species (ROS), reactive nitrogen species, and endogenous antioxidant defenses is a central contributor to the onset and progression of metabolic and degenerative diseases. Elevated ROS levels are implicated, for example, in diabetes, obesity, diabetic nephropathy and neuropathy, cardiovascular disorders, and neurodegenerative diseases such as Alzheimer’s disease and Parkinson’s disease [3, 4]. Excess ROS also promotes lipid peroxidation, protein carbonylation, and nucleic acid damage, ultimately impairing cellular function and triggering inflammation. While mitochondria represent the primary endogenous source of ROS through electron transport chain activity, oxidative stress can also arise from environmental and dietary factors.

Food-derived peptides offer a promising natural strategy to neutralize reactive species and mitigate oxidative damage [5]. Numerous studies have demonstrated inverse associations between antioxidant intake and oxidative stress-related diseases [3, 6, 7]. The antioxidant activity of peptides is mediated through multiple complementary mechanisms, including free radical scavenging, metal ion chelation, inhibition of lipid peroxidation, and modulation of intracellular redox-regulating pathways. These pathways encompass cytoprotective signalling, upregulation of endogenous antioxidant enzymes, and suppression of pro-inflammatory mediators. Their efficacy is strongly influenced by amino acid composition and structural features, with residues such as cysteine (C), histidine (H), tyrosine (Y), tryptophan (W), and phenylalanine (F) conferring enhanced redox activity [5, 8]. They are often viewed as safer and more biocompatible alternatives to synthetic antioxidants which, while effective, have raised toxicological concerns related to cytotoxicity, endocrine disruption, and carcinogenic potential when consumed at high levels or over prolonged periods [9]. Peptides, conversely, are naturally occurring, biodegradable, exhibit lower risk of accumulation in tissues, and offer multifunctional health benefits beyond antioxidation. As a result, they are being widely explored as additives to prevent oxidative deterioration in foods and as therapeutic agents to mitigate oxidative stress-related diseases [1, 8].

Despite these advantages, important gaps remain in understanding the safety of antioxidant peptides, particularly regarding structural changes and potential toxicities arising from oxidative modifications, which refers to the alteration of peptide or protein structures through reactions with ROS or secondary oxidation products. Peptides are active participants in redox reactions and can undergo substantial chemical transformations during ROS neutralization [10]. These include the formation of peptide hydroperoxides, carbonyl derivatives, alcohols, backbone fragmentation products, and cross-linked dimers such as dityrosine. Additionally, their interactions with reactive aldehydes generated during lipid oxidation (e.g., malondialdehyde, 4‑hydroxy‑2‑nonenal) or with reducing sugars through glycation can yield Schiff bases (imine linkages between carbonyls and amines), Michael adducts (nucleophilic additions to α,β‑unsaturated carbonyls), and advanced lipoxidation or glycoxidation products (stable cross‑linked structures formed from oxidized lipid- or sugar-derived intermediates) [11, 12]. In proteins, such oxidative modifications are associated with reduced digestibility, altered allergenicity, membrane-disruptive behaviour, and cytotoxicity [13]; however, analogous consequences for short peptides remain insufficiently characterized. Given the growing incorporation of bioactive peptides into functional foods and nutraceutical formulations, a comprehensive evaluation of their safety is essential. Key considerations include identifying concentrations at which antioxidant peptides may exhibit toxicity, understand the biological behaviour of their oxidative metabolites, and determining whether structural modifications influence metabolic responses. Although antioxidant peptides are often considered safer alternatives to synthetic antioxidants and have demonstrated protective effects against chronic diseases [1], their potential toxicity, particularly at high doses or in their in-situ modified forms requires careful assessment.

Existing reviews have mostly focused primarily on the bioactivity and mechanisms of antioxidant peptides, with limited attention to their safety profiles. Therefore, this review aims to integrate current knowledge on the mechanisms underlying the antioxidant activity of food-derived peptides while introducing safety assessment as a critical dimension for their responsible use. By examining both their beneficial actions and potential toxicological outcomes, this work provides a balanced foundation for advancing the safe and effective application of antioxidant peptides in food systems and health-promoting products.

## Bioactive food-derived peptides and safety during production

Bioactive food peptides, short amino-acid sequences, encrypted within parent food proteins and released upon proteolysis, have attracted considerable scientific attention due to their diverse physiological effects, including antioxidant, antihypertensive, immunomodulatory, antimicrobial, and metabolic regulatory activities [14, 15]. Their functionality depends on intrinsic structural features such as amino-acid composition, sequence, hydrophobicity, electronic properties, molecular weight, and secondary conformation, as well as on the method of production and the characteristics of their protein precursors [16, 17]. These peptides play an important and growing role in food science and human health, as they offer natural, multifunctional bioactivities that extend beyond the nutritional value of whole proteins.

### Sources and production

The peptides are obtained from a wide spectrum of edible proteins, sourced from both plant and animal systems. Plant-derived proteins from legumes, cereals, oilseeds, roots, and leafy greens provide abundant reservoirs of latent bioactive sequences. Likewise, animal proteins, including those from by-products of meats and fish, dairy, eggs, and, more recently, insects, serve as accessible precursors [18, 19]. The structural diversity among these proteins contributes to the broad repertoire of bioactive sequences found in hydrolysates across food systems and after purification of the hydrolysates. The release of bioactive peptides from these precursor proteins occurs primarily through proteolytic mechanisms. Classical production approaches rely on enzymatic hydrolysis using digestive enzymes (e.g., pepsin, trypsin, chymotrypsin), or food-grade enzymes such as alcalase, flavourzyme, and papain. Microbial fermentation represents another major route of peptide production, wherein endogenous proteases from bacteria, yeasts, or fungi gradually liberate peptides during the fermentation process [20]. A less frequently used method is autolysis or self-degradation by endogenous proteases (i.e. generation of bioactive peptides without the addition of external enzymes) [21]. The specificity of the protease, the hydrolysis conditions, and the structural accessibility of cleavage sites together determine the peptide profiles formed and ultimately their bioactivities.

**Fig. 1:**
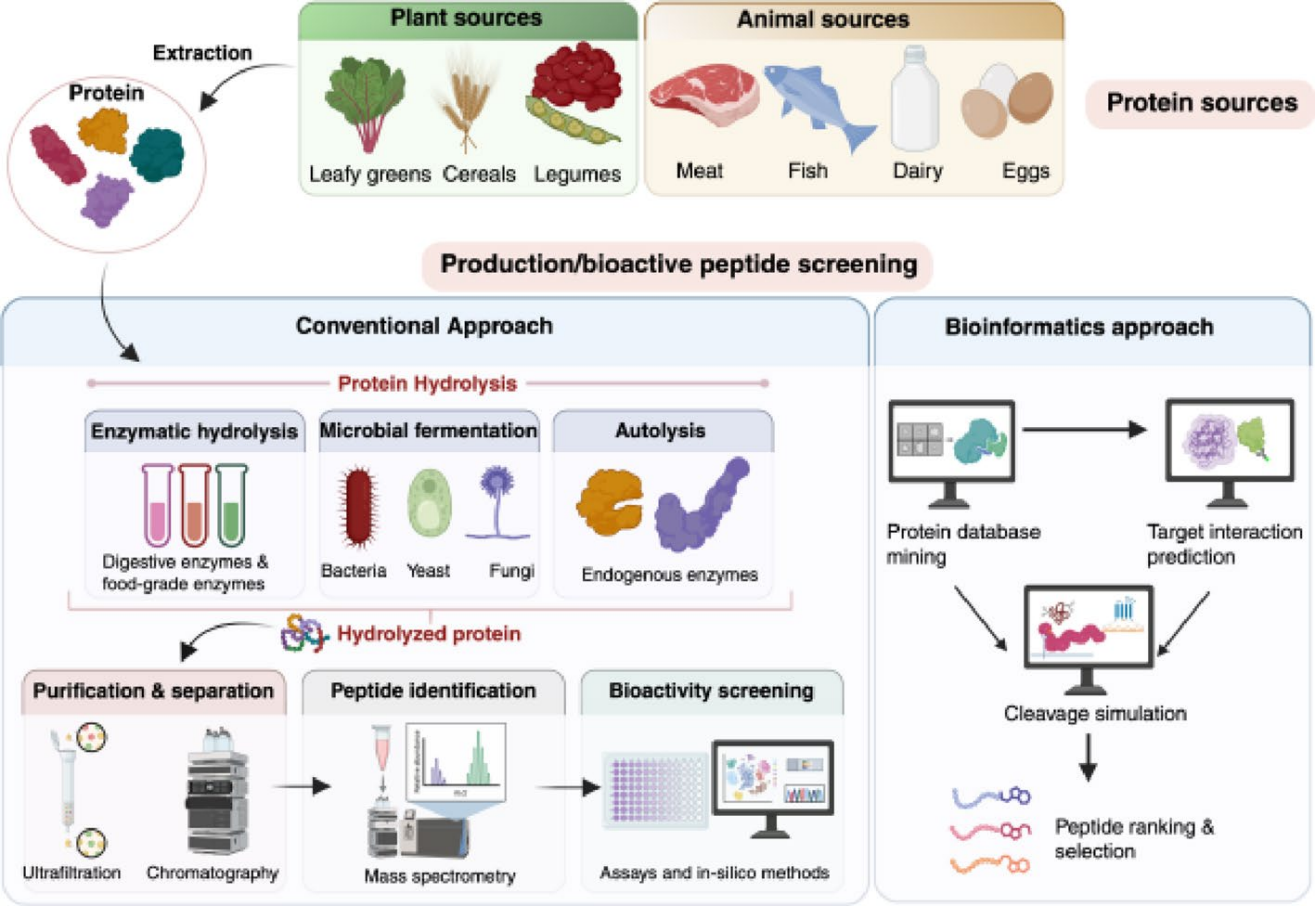
Production of peptides from plant and animal sources. Created in BioRender. Mylvaganam, H. (2026) https://BioRender.com/pgwcg8a

Beyond biochemical production methods, bioactive peptide discovery has increasingly incorporated computational strategies [22, 23]. Two complementary frameworks exist (Fig. 1): the classical workflow and the bioinformatics-driven approach. The classical process involves protein extraction, enzymatic hydrolysis, sequential purification (ultrafiltration, chromatography), bioactivity screening, and peptide identification using mass spectrometry [24]. This empirical workflow remains essential for validating functional peptides and characterizing their structures. In contrast, the bioinformatics approach accelerates discovery by applying in silico tools to mine protein databases for encrypted bioactive motifs, predict peptide target interactions, simulate protease cleavage patterns, and prioritize candidate sequences for synthesis [23]. This approach substantially reduces the time and cost associated with peptide discovery and allows researchers to focus on sequences that exhibit high predicted functionality before experimental testing. The integration of classical and computational strategies has become increasingly important for systematically identifying and optimizing food bioactive peptides for food and nutraceutical applications [25]. Bioinformatics tools can then accelerate peptide discovery and predict potential bioactivities; meanwhile, most in silico protease-cleavage models evaluate a single protein, assuming uniform accessibility of peptide bonds and the absence of structural constraints. In contrast, protein isolates contain complex mixtures of proteins, many of which compete or interact during hydrolysis and may alter protease specificity. As a result, the predicted peptides can differ substantially from the peptides generated experimentally in real hydrolysates.

Bioactive peptides can undergo chemical transformations during their release, purification, or storage (e.g. oxidation, glycation, lipoxidation, aggregation, and Maillard-type reactions), making it essential to evaluate their safety independently of the production method. Accordingly, both conventional enzymatic or fermentation-based approaches and bioinformatic discovery pipelines require downstream assessment of peptide stability, the potential formation of harmful reaction products, allergenic risks, and metabolic fate to ensure that the resulting compounds remain safe for use in foods and related products.

### General characteristics of food-derived peptides

Food bioactive peptides share several structural and physicochemical characteristics that influence their biological effects, stability in food matrices, behaviour during digestion, and applicability in functional foods. Amongst the major biological functions of peptides, their antioxidant activity is the most widely studied and mechanistically well-supported. Nevertheless, the same physicochemical determinants that underlie antioxidant activity also contribute to other functional roles. Features that shape peptide activity include molecular weight, typically in the range of 250–1800 Da [26]. The smaller peptides can display superior bioactivity due to enhanced solubility, reduced steric hindrance, and better tissue permeability. They can diffuse to target sites, interact with radicals or metal ions, and a certain percentage could be absorbed across intestinal epithelia. Molecular weight influences not only bioactivity but also techno-functional properties such as emulsification, solubility, and foaming. Another determinant is amino-acid composition and sequence [27, 28]. The peptides enriched in specific amino acids exhibit enhanced functional properties. Histidine-containing peptides frequently show high antioxidant activity due to the imidazole ring’s ability to participate in proton-coupled electron transfer and metal chelation. Tyrosine and tryptophan contribute hydrogen-donating phenolic and indole groups, stabilizing the corresponding radicals via resonance. Sulfur-containing residues, including cysteine and methionine, serve as potent electron donors and redox-active centers, enabling peptides to reduce peroxides or quench reactive intermediates [27, 29]. Moreover, acidic amino acids such as aspartate and glutamate act as metal chelators, suppressing Fenton chemistry by binding Fe^2+^ and Cu^2+^ [8]. The sequence position of these residues is equally important; hydrophobic residues (Leu, Val, Pro), when present at the N-terminus, increase interfacial affinity, while central aromatic residues maximize hydrogen-donating efficiency. These patterns underscore the intimate relationship between sequence architecture and antioxidant potency.

Hydrophobicity is another property, as hydrophobic peptides tend to partition into lipid-rich phases, enabling them to quench lipid peroxyl radicals more effectively. This interfacial positioning is particularly important in food systems such as emulsions, where lipid oxidation occurs at droplet surfaces [30]. Hydrophobicity also enhances interactions with cellular membranes, potentially affecting peptide uptake, stability, and biological performance [28, 29]. Hydrophobicity also contributes to functional properties by influencing solubility, emulsification, and aggregation, ultimately determining how peptides behave within food matrices, all of which may affect their antioxidant activities.

Peptide charge and isoelectric point (pI) influence solubility, interactions with radical species, and affinity for metal ions, with acidic residues typically enhancing metal chelation and basic residues favouring binding to negatively charged radicals or lipid headgroups; these electrostatic properties also shape interactions with proteins and polysaccharides in food matrices, ultimately affecting peptide stability, availability, and antioxidant performance [31, 32]. Solubility and stability further determine how peptides behave under processing conditions, with highly soluble sequences being able to disperse more effectively in aqueous systems and exhibit improved bioavailability, while resistance to heat, oxidation, and pH shifts, as well as stability during digestion, contribute to whether peptides reach target tissues intact or rely on subsequent enzymatic cleavage for bioactivity. Structural features add another layer of modulation, as secondary structure and conformational flexibility affect the accessibility of reactive groups. Flexibility, for example, can more readily expose electron‑donating residues, whereas transiently folded motifs may shield reactive amino acids from premature oxidation or degradation, collectively shaping the overall antioxidant potential of the peptide. Taken together, the general characteristics of food bioactive peptides reflect a complex interplay between intrinsic physicochemical properties and their behaviour in both food and physiological environments. The contribution of these properties to the bioactivity of peptides underscores the importance of rational design and targeted production strategies to maximize their functional potential.

## Mechanisms of antioxidant activity and mechanism of food-derived peptides

**Fig. 2:**
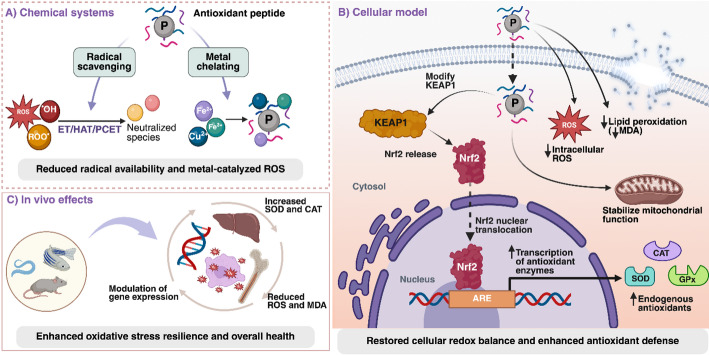
Sketch illustrating the mechanism of antioxidant peptides in chemical systems (**A**), cellular models (**B**) and animal models (**C**). Electron transfer (ET); hydrogen atom transfer (HAT), proton-coupled electron transfer (PCET). Created in BioRender. Mylvaganam, H. (2026) https://BioRender.com/19rbnxs

Food-derived antioxidant peptides counter oxidation through a combination of chemical and biological mechanisms that are consistently observable across model systems ranging from cell-free redox assays to cultured cells and in vivo studies as illustrated in (Fig. 2). In chemical systems (Fig. 2A), peptides directly scavenge radical species by donating electron or hydrogen and reducing or chelating transition metals that otherwise catalyze ROS formation [5, 33]. In cellular models (Fig. 2B), the peptides often lower intracellular ROS, reduce lipid peroxidation markers, and modulate the activity of endogenous antioxidant enzymes such as superoxide dismutase (SOD), catalase (CAT), and glutathione peroxidase [34]. In vivo (Fig. 2C), peptide administration has been associated with restored redox balance in tissues, protection against oxidation of biomolecules, improved mitochondrial integrity, and attenuation of inflammation-associated oxidative damage [5, 29]. Through these multipathway interactions, antioxidant peptides exert protective effects in biological and food systems.

### Radical scavenging activity

The most commonly assessed property of antioxidant molecules, including food-derived peptides, is their capacity to quench oxidants, particularly radical species. Direct quenching is therefore a defining feature of many antioxidant peptides and occurs through electron transfer (ET), hydrogen atom transfer (HAT), or proton-coupled electron transfer (PCET) (Fig. 2A). Chemical assays widely used to characterize these reactions reflect their underlying mechanistic pathways. The oxygen radical absorbance capacity (ORAC) assay predominantly captures HAT mechanisms, since peptides protect a fluorescent probe from peroxyl radicals generated by AAPH. In contrast, DPPH and ABTS•+ are historically interpreted as ET-based assays, although mounting evidence shows that both radicals can also be neutralized through HAT pathways.

These mechanistic interpretations are supported by extensive testing of individual peptides. For example, antioxidant peptides isolated from pea protein hydrolysates YLVN, EEHLCFR, and TFY demonstrate strong performance in all three assays [35]; YLVN exhibits exceptionally high ABTS activity (IC_50_ = 0.002 mg/mL) and ORAC values (1.12 ± 0.23 µmol TE/µmol), outperforming glutathione, while EEHLCFR shows potent DPPH scavenging (IC_50_ = 0.027 mg/mL). Similarly, whey protein-derived peptides such as LDQW, YW, WYS, GYDTQ, and WY display radical-scavenging capacities, with LDQW demonstrating the highest ORAC activity (3.79 ± 0.15 µmol TE/µmol) and YW and WYS showing the highest scavenging capacities for DPPH (22.5% ± 1.8%) and ABTS (1.3 ± 0.1 mM TE/mM peptide) radicals, respectively [36]. Broader screening studies of 36 dipeptides confirmed the complementary activities of different scavenging tests and the contribution of sequences containing amino acids capable of donating electron or proton to the scavenging peroxyl, DPPH, and ABTS radicals [37].

The chemical feasibility of radical quenching aligns with the presence of amino acids whose oxidized forms are resonance-stabilized or otherwise low-energy. Cysteine undergoes thiol-sulfenyl chemistry, tyrosine generates stable phenoxyl radicals, tryptophan disperses charge across the indole ring, histidine stabilizes imidazolyl radicals, and methionine or phenylalanine provide thioether and aromatic stabilization [8]. Even proline, although not aromatic, supports α-carbon abstraction and contributes uniquely to HAT chemistry. These are exemplified by peptides such as Phe-Cys (FC) derived from microalgal RuBisCO protein that protected liver cells through rapid hydrogen donation from the Cys thiol. Peptides such as RDY from mulberry leaf proteins, SPFWNINAH from oat proteins, displayed strong DPPH, ABTS, or peroxyl radical scavenging activities [14, 38] which can be attributed to the readiness of phenoxyl and indole hydrogen to participate in HAT or PCET. The higher radical scavenging activities of bean proteins derived peptides, TETWNPNHPEL ( ABTS EC_50_ = 0.5 ± 0.2 mM and DPPH EC_50_ = 2.1 ± 0.1 mM), and TETWNPNHPE (ORAC 2.84 ± 0.08 mM Trolox equivalent/mM) [39] can be attributed to a combination of HAT, ET, and PCET mechanisms related to the presence of Trp (W), Tyr (Y), and His (H), although the contribution of Pro, Glu, and Asp should not be neglected. Tyr-bearing peptides such as Tyr-Leu (YL) and Phe-Tyr (FY) perform well in ORAC, DPPH, and ABTS assays and maintain a good activity in hepatocyte models [40], directly linking phenolic chemistry to biologically relevant antioxidant behaviour. Together, these findings demonstrate that side-chain chemistry, particularly the presence and accessibility of electron-rich residues, rather than peptide length alone, governs radical-scavenging efficiency across chemical, cellular, and physiological contexts. Table 1 provides examples of antioxidant peptides, their activities and the contribution of amino acids.

**Table 1:** Structure-activity relationships of food-derived antioxidant peptides

Peptide (source)	Structural features	SAR interpretation	Antioxidant activity
SDGSNIHFPN (Snakehead *Channa argus*)	Contains His, acidic residues (Asp/Glu), flexible backbone	His enables metal chelation; acidic residues provide bidentate Fe^2+^ binding	High Fe^2+^ chelation: IC_50_ = 4.60 mM [17]
IVLPDEGK, SVSIRADGGEGEVTVFT (Snakehead)	Asp/Glu-rich sequences	Chelation dominated by acidic residues with electrostatic stabilization	Fe^2+^ chelation IC_50_ = 7–26 mM [17]
GHHAAA, PHPR, SVTEV, VRDQY, SMDV (Skipjack tuna milt)	His-rich, aromatic (Tyr), redox-active residues	His → metal coordination; Tyr → radical stabilization	Strong ferric-reducing power (dose-dependent) + cytoprotection in HUVECs [41]
QQPQPW (Corn protein)	Terminal Trp, multiple Gln/Pro	Trp contributes indolic H-donation; Pro increases flexibility	Moderate Fe^2+^ chelation EC_50_ = 6.27 mg/mL [42]
CSQAPLA (Corn)	Reactive Cys at N-terminus	Cys thiol → potent electron donor	High reducing power IC_50_ = 0.116 mg/mL [43]
WVYY (Hemp seed)	Trp + Tyr–Tyr aromatic triad	π-systems facilitate resonance stabilization; multi-aromatics increase reducing/chelating power	94–96% Fe^2+^ chelation at 0.5 mg/mL [44]
PSLPA (Hemp seed)	Pro/Leu hydrophobic motif	good lipid-interfacial activity	96% Fe^2+^ chelation at 0.5 mg/mL [44]
AVPYPQR (Casein)	Tyr–Pro aromatic core + Arg	Tyr for H-donation; Arg stabilizes binding	59.76% Fe^2+^ chelation at 1 mg/mL (highest among casein peptides) [45]
HKEMPFPK (Casein	Met + Phe aromatic cluster	Met sulfur → reducing activity; aromatic cluster stabilizes radicals	Fe^2+^ chelation 8.02% at 1 mg/mL [45]
VYLPR, EVYLPR (Egg white)	Multiple aromatics + acidic/basic balance	Aromatic residues provide quenching; Lys/Arg modulate solubility & binding	Reduction of ROS + protection of HEK-293 cells [46]
LALPVYN (Moringa leaves)	Hydrophobic cluster + Tyr	Hydrophobic interface targeting increases access to lipid radicals	Significant ROS/MDA reduction; ↑SOD, CAT, GSH-Px in HepG2 cells [47]
DTYIRQPW, WDDMEKIWHH (Duck liver)	Tyr + Trp + His clusters	Synergistic aromatic + imidazole radical chemistry	Cytoprotection vs. H_2_O_2_ in HepG2 cells; restores antioxidant enzymes [34]
FC (Microalgal RuBisCO)	Aromatic + thiol	Phe increases lipid interaction; Cys offers powerful thiol-based redox capacity	Reduces intracellular ROS by up to 45%; inhibits lipid peroxidation 35–61% [48]
LPGYF (Tilapia)	Pro-Gly flexibility + Tyr/Phe aromatics	Pro enhances interfacial affinity; Tyr/Phe donate H atoms	Highest ROS scavenging in AAPH-treated HepG2 cells [49]
VENAACTTNEECCEKK, VEGGAACTTGGEEGCCEKK (*Arca subcrenata*)	Multiple Cys, acidic residues	Multi-Cys = strong redox/chelating behaviour	Extend C. elegans lifespan; ↓ROS, ↓lipofuscin accumulation [50]

### Metal chelation activities of antioxidant peptides

Transition metals such as Fe^2+^, Fe^3+^, and Cu^2+^ are central catalysts of oxidative deterioration through Fenton and Haber-Weiss reactions, where Fe^2+^ reacts with hydrogen peroxide to generate highly reactive hydroxyl radicals (Fig. 2A). These radicals accelerate lipid rancidity, pigment and nutrient loss, and toxic oxidation product formation in foods, while in biological systems they promote biomolecule oxidation and cell damage [33]. Antioxidant peptides counter these metal‑driven processes primarily by chelating Fe^2+^/Fe^3+^ and Cu^2+^, thereby reducing their catalytic availability, and by exhibiting ferric‑reducing activity that converts Fe³⁺ into less reactive forms. Together, these mechanisms suppress the initiation and propagation of oxidative reactions in food matrices and cellular environments. Across animal, plant, and dairy proteins, numerous peptides have demonstrated strong metal‑chelating capacity (Table 1). Peptides from Channa argus (snakehead), for example, show notable Fe^2+^ chelation, with SDGSNIHFPN exhibiting the highest activity (IC_50_ = 4.60 ± 0.05 mM) and others such as IVLPDEGK and SVSIRADGGEGEVTVFT displaying IC_50_ values of 7–26 mM [17]. Similar metal‑related antioxidant mechanisms are observed in skipjack tuna milt peptides GHHAAA, PHPR, SVTEV, VRDQY, and SMDV, which display concentration‑dependent ferric‑reducing power [41], reflecting their ability to deactivate metals through redox modulation.

Plant-derived peptides also contribute significantly to metal chelation. Corn protein hydrolysates yield sequences such as QQPQPW, which showed moderate Fe^2+^ chelation (EC_50_ = 6.27 mg/mL), while CSQAPLA exhibits a stronger ferric‑reducing activity (IC_50_ = 0.116 mg/mL), underscoring the role of cysteine thiolate chemistry [42, 43]. Hemp seed peptides WVYY and PSLPA further illustrate potent chelation, binding 94% and 96% of Fe^2+^ at 0.5 mg/mL, respectively, due to the synergistic contributions of Trp (W), Tyr (Y), and Pro (P) in stabilizing metal–peptide complexes [44]. Dairy proteins provide additional examples: casein‑derived peptides EDVPSER, HKEMPFPK, NMAINPSK, and AVPYPQR exhibit Fe^2+^ chelation ranging from 7.57% to 59.76% at 1 mg/mL, with AVPYPQR showing the highest activity, consistent with its combination of acidic, aromatic, and basic residues that enable multidentate metal binding [45]. Collectively, the literature data highlight that peptide metal‑chelating capacity is correlated to the overall structure and the proper positioning of ligating groups such as imidazole, thiolate, carboxylates, and ε‑amine capable of forming coordinate bonds with metal ions, while aromatic residues could stabilize complexes through π‑electron interactions. Peptides containing multiple coordinating residues often form bi‑ or multi-dentate complexes with transition metal ions, markedly reducing their reactivity and the formation of hydroxyl radicals.

### Activity of antioxidant peptides in cellular and animal models

Antioxidant peptides have demonstrated activities across cellular, invertebrate, and mammalian systems, where they attenuate oxidative stress through a network of interconnected mechanisms. These pathways include reducing intracellular ROS, inhibiting lipid peroxidation, and restoring the balance of endogenous antioxidant enzymes [34]. They also help stabilize mitochondrial function, by regulating the activity of nicotinamide adenine dinucleotide phosphate (NADPH) oxidase as well as activating redox‑responsive signalling cascades such as the Kelch‑like ECH‑associated protein 1 (Keap1), nuclear factor erythroid 2–related factor 2 (Nrf2), and antioxidant response element (ARE) axis [51]. Their mechanisms are summarized in Fig. 2B and C. In cultured cells, a diverse range of peptides from animal, plant, marine, and microbial proteins show cytoprotective effects (Table 1). For example, a yak bone collagen-derived peptide (GGGPPGPM) reduced UVA-induced damage in human fibroblasts by lowering MDA levels while restoring antioxidant enzymes (CAT and SOD) activities, and exerted its effects through activation of the Keap1/Nrf2 pathway, which increased expression of Nrf2, heme oxygenase, and NADPH-quinone oxidoreductase by 19.57%, 41.41%, and 34.94%, respectively [52]. Similar protective behaviour is reported for Moringa oleifera leaf peptide LALPVYN, which enhanced HepG2 cell viability, increased CAT, SOD, and GSH-Px activities, and decreased ROS, MDA, and apoptosis under oxidative challenge [47]. Duck liver peptides (DTYIRQPW, WDDMEKIWHH) and aromatic-rich perilla seed peptides (YL, FY) similarly mitigated H_2_O_2_‑induced oxidative stress in HepG2 cells by enhancing antioxidant enzyme activities and suppressing lipid peroxidation, with the latter also protecting rat liver homogenates by reducing lipid peroxidation [34, 40]. The microalgal RuBisCO-derived dipeptide (FC) produced dose-dependent inhibition of intracellular ROS in Chang liver cells, reducing H_2_O_2_-induced ROS by 20–45% and lipid peroxidation by up to 61%, while boosting antioxidant enzyme levels more than two-fold [48]. Additional protective responses have been observed with tilapia peptides (e.g., LPGYF, PGY), hazelnut peptides (EW, ADGF, DWDPK), cottonseed meal peptide LGSPDVIVIR, porcine plasma peptide EDEQKFWGK, and krill peptides LKPGN and LQP, all of which increased SOD, CAT, or GSH-Px activities and decreased ROS, MDA, and nitric oxide-derived superoxide anion to maintain cellular redox balance [16, 47, 49].

The antioxidant activity of peptides extends beyond cell culture and has been demonstrated in invertebrate models such as *Caenorhabditis elegans*. Arca subcrenata peptides VENAACTTNEECCEKK and VEGGAACTTGGEEGCCEKK significantly extended mean lifespan under paraquat-induced oxidative stress, reduced whole-organism ROS, fat, and lipofuscin accumulation, and improved age-associated physiological performance [50]. Transcriptomic analyses showed that these peptides downregulated the aging-associated gene *age-1* while upregulating multiple stress-responsive genes [50], indicating that peptide-induced redox protection operates partly through genetic pathways that regulate energy metabolism and stress resilience. Yak bone–derived peptides GASGPMGPR and GLPGPM further confirmed these systemic antioxidant effects in *C. elegans* by lowering ROS and MDA levels while elevating SOD and CAT activities, collectively contributing to extended lifespan and improved oxidative stress tolerance [53]. In mammalian models, antioxidant peptides have demonstrated protective effects in liver, bone, and developmental oxidative stress conditions. The casein-derived peptides VLP and VLPVPQK respectively, alleviated CCl₄‑induced oxidative liver injury in mice via activation of the KEAP1-NRF2-ARE pathway and broad-spectrum osteoprotective effects in ovariectomized rats by enhancing antioxidant defenses (SOD, CAT, GSH), lowering MDA, improving bone mineral density and trabecular architecture, strengthening bone, and suppressing bone‑resorbing cytokines [51, 54]. Marine peptides also exhibit in vivo antioxidant potential; in zebrafish embryos exposed to H_2_O_2_, C-phycocyanin–derived peptides MHLWAAK, MAQAAEYYR, and MDYYFEER markedly suppressed ROS and MDA formation while enhancing SOD and CAT activity [55], demonstrating their ability to counteract oxidative teratogenesis during development. These data are evidence of antioxidant peptides exerting multifaceted biological effects that translate across model systems to whole organisms. Despite the growing number of studies demonstrating the antioxidant activity of peptides in cellular and in vivo models, an important limitation remains in attributing these effects to the intact peptide structures or their digestion-derived fragments. Given that intestinal absorption is largely limited to peptides containing 2–3 amino acids, future studies should combine activity with bioavailability.

## Structural changes of peptides during antioxidant action and safety of the products

**Fig. 3:**
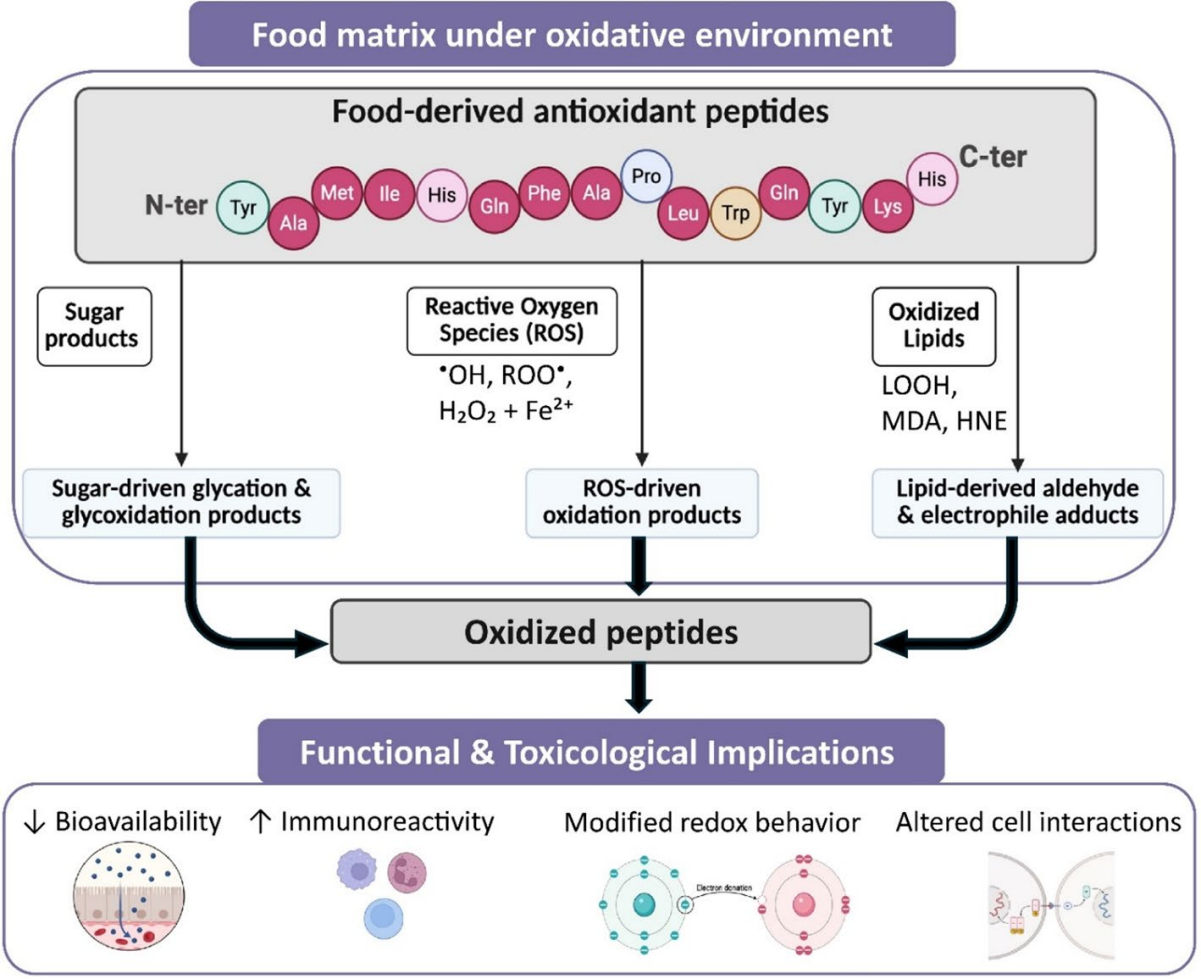
Oxidative transformations of food-derived antioxidant peptides and resulting biological effects

Antioxidant food-derived peptides do not merely neutralize oxidants; they are themselves transformed through a network of radical and carbonyl driven reactions [8, 16]. As these peptides encounter hydroxyl, peroxyl, and other reactive species, they form a spectrum of oxidation products, including hydroperoxides, alcohols, carbonyls, and peptide backbone fragments. Covalent modifications can also arise through reactions with aldehydes derived from lipids and sugars, generating Schiff-base and Michael-type adducts, as well as advanced lipoxidation and glycoxidation end-products [12, 56, 57]. In addition to these small-molecule adducts, certain oxidative pathways promote amino acid dimerization, such as di-tyrosine cross-links, or other residue-specific dimeric structures that irreversibly alter peptide architecture and potential bioactivity [8]. The formation of oxidized food-derived peptides and their functional and safety implication are illustrated in Fig. 3. While the detrimental consequences of oxidative products have been extensively documented in proteins, where they contribute to aggregation, loss of solubility, altered allergenicity, membrane-disruptive behaviour, and impaired enzymatic or structural function [13, 58, 59], the biological implications of analogous modifications in bioactive peptides remain largely unexplored. The formation of peptide hydroperoxides, cross-linked dimers, and electrophilic adducts may attenuate or redirect their physiological roles or introduce entirely new bioactivities with unrecognized safety liabilities. This knowledge gap underscores the need for systematic structural-functional studies to understand how oxidation transforms peptide-based ingredients intended for health and nutrition applications. This gap highlights the need for researchers to identify the oxidative products of antioxidant peptides, elucidate their mechanism of formation, and determine their functions under diverse conditions to ensure their safe use in food and related systems. A structure-toxicity relationship framework for oxidized peptide products is proposed (Table 2). The framework highlighted the modification type, structural features, dominant chemical reactivity, potential biological or toxicological effects, and the assessment that could be performed.

**Table 2:** Proposed Structure–toxicity relationships of oxidized food-derived peptide products

Oxidative modification type	Key structural features	Dominant chemical reactivity	Representative formation pathways	Potential biological/toxicological effects	Implications for safety assessment
Hydroperoxides (–OOH)	–OOH on side chains (e.g., Trp, Tyr, Pro, His)	decomposition into RO•/ ROO• radicals	ROS-mediated oxidation metal-catalyzed H_2_O_2_ systems	Propagation of oxidative stress; lipid peroxidation; membrane damage	Assess stability, decomposition kinetics, evaluate ROS amplification potential
Carbonyl derivatives (aldehydes, ketones)	Backbone fragmentation products, deamination	Reactivity toward nucleophiles (e.g., amines, thiols)	Secondary oxidation of amino acids, ROOH breakdown	Protein/DNA adduct formation, cytotoxicity, genotoxicity	Quantify carbonyl content; evaluate adduct formation and persistence
Covalent dimers (e.g., dityrosine)	Cross-linked aromatic residues; increased molecular weight	Radical coupling, reduced structural flexibility	Tyrosyl radical recombination under oxidative conditions	Altered function, aggregation; reduced digestibility	Assess digestibility, absorption, and potential accumulation
Lipid-derived aldehyde adducts (MDA, HNE)	Schiff base or Michael adducts	Nucleophile–electrophile reactions with Lys, His, Cys, Arg	Conjugation of peptides with MDA, HNE	Membrane disruption, mitochondrial dysfunction, inflammation, cytotoxicity	Identify adducts via LC–MS; evaluate membrane interaction and cellular toxicity
Pyrrole and cyclic lipoxidation products	Aromatic/cyclic structures derived from aldehyde condensation	Increased stability and lipophilicity, membrane interaction	Advanced lipoxidation reactions (e.g., HNE-derived pyrroles)	Membrane insertion, altered permeability, oxidative signalling disruption	Assess bioaccumulation potential and interaction with lipid bilayers
Glycated peptides (early Maillard products)	Schiff bases and Amadori intermediates	Moderate reactivity; reversible covalent bonding	Reaction with reducing sugars under mild conditions	Altered antioxidant activity; variable metabolic effects	Evaluate stability and reversibility; assess in vivo relevance
Advanced glycoxidation end-products (AGEs)	Crosslinked, fluorescent, heterogeneous structures	Strong receptor binding, oxidative amplification	Advanced Maillard reactions under heat/oxidative conditions	Pro-inflammatory signalling, oxidative stress induction, chronic toxicity risks	Quantify AGE markers; evaluate receptor-mediated toxicity pathways
Heterocyclic reaction products (e.g., HAAs, acrylamide-related systems)	Aromatic heterocycles formed under high-temperature conditions	Interaction with DNA and proteins; electrophilic reactivity	Thermal processing (Maillard reaction, pyrolysis)	Mutagenicity, carcinogenic potential	Monitor processing conditions; quantify formation and exposure risk

### Oxidative modification of bioactive peptides by reactive oxygen species

**Fig. 4:**
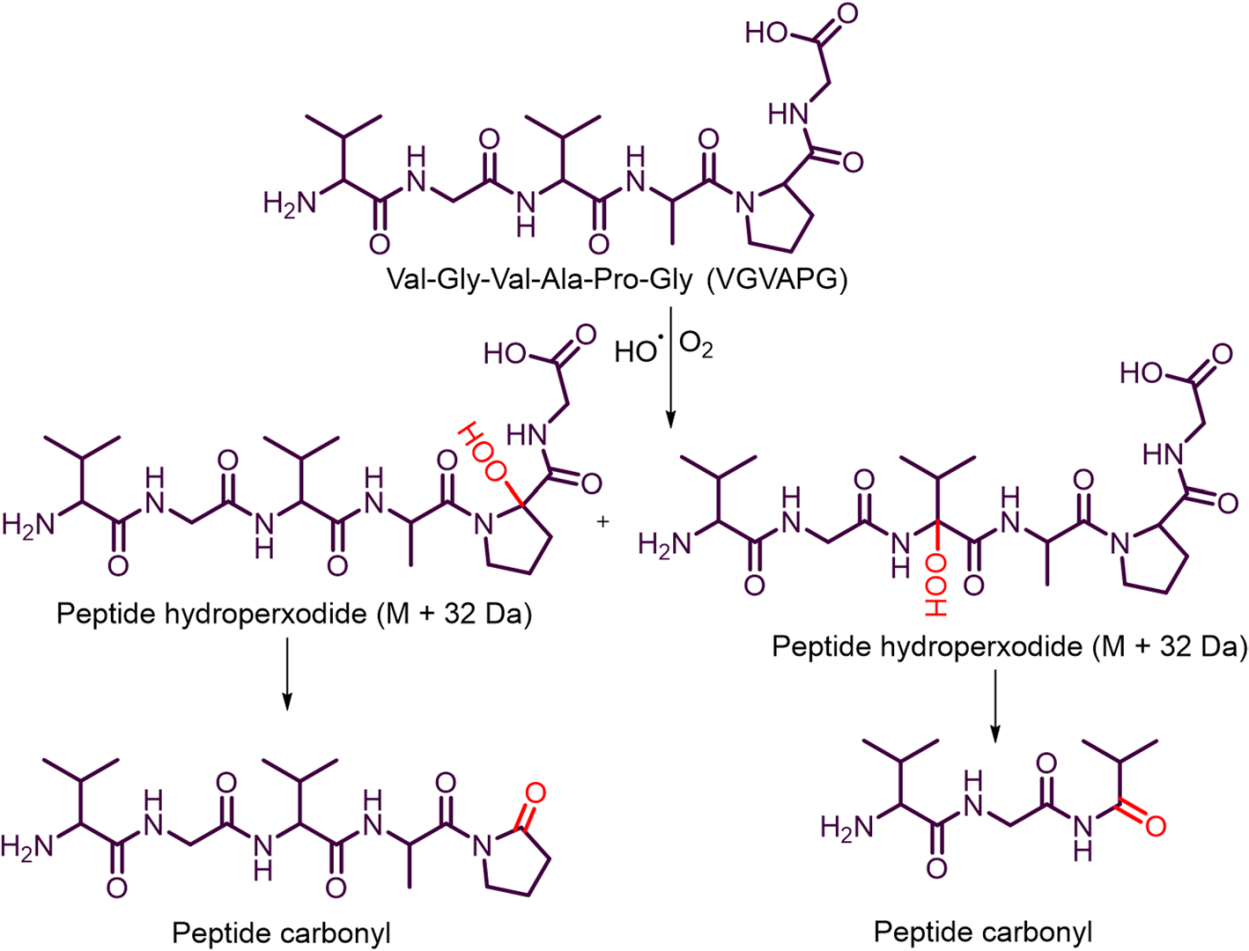
Hydroperoxides and carbonyls from oxidized peptide VGVAPG

Reactive oxygen species, including hydroxyl radicals (•OH), hydrogen peroxide (H_2_O_2_), and peroxyl radicals (ROO•) are capable of inducing structural modifications to food-derived peptides. Hydroxyl radicals, generated either directly or from H_2_O_2_ through metal-catalyzed Fenton chemistry, initiate site-specific oxidation leading to peptide hydroperoxides (POOH + 32 Da), alcohols (+ 16 Da), carbonyls (+ 14 Da), backbone scission products, and amino-acid-specific dimerization events [10, 60]. In a model system, the scavenging of HO^•^/O_2_ by peptide Val-Gly-Val-Ala-Pro-Gly yielded different oxidation products (alcohols, carbonyls, hydroperoxides, fragment species) [60]. Mass spectrometry data showed hydroperoxides were present as a mixture in which every amino acid was oxidized. The mass of each fragment was shifted by + 32. The proposed hydroperoxide structures are shown in Fig. 4.

**Fig. 5:**
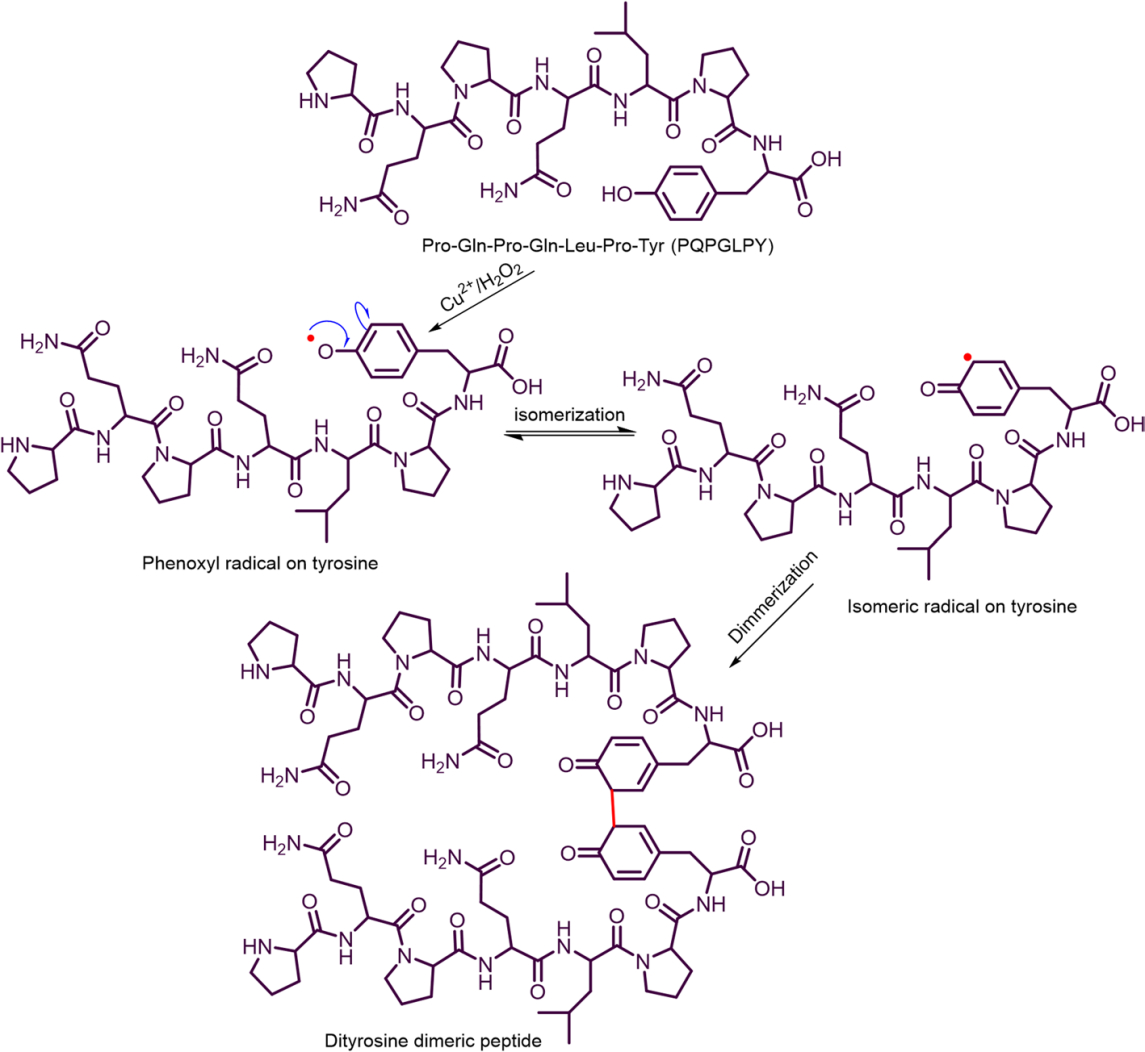
Example of oxidized Cu^2+^/H_2_O_2_ oxidative products of peptide PQPQLPY

The QQPFP and PQPQLPY from barley proteins in the presence of HO• radicals from a Cu/H_2_O_2_ system also produced peptide hydroperoxides, carbonyls, in addition to a dimer form from covalent linking of the tyrosine residues [61]. The dityrosine adduct could be formed from PQPQLPY as illustrated in Fig. 5. Tryptophan-containing antioxidant peptides can also undergo ROS-driven transformations. In the presence of O_2_•– and ROO• radicals, di- and tri-peptides (GW, WG, GWG, WA, and AWA) yielded Trp-derived hydroperoxides, which subsequently decompose to N-formylkynurenine (+ 32 Da), kynurenine (+ 4 Da), multiple Trp-alcohols, and ring-opened species [62, 63]. In a related work, antioxidant peptides SPFWNINAH, NINAHSVVY from oat proteins in the presence of ROO• lost more than 50% of their concentrations as they were oxidized to mono and di-hydroxylated derivatives [10].

Across all ROS pathways, oxidative products and covalent oxidative dimerization contribute to structural rearrangement, aggregation, and altered physicochemical behaviour. The consequences of these reaction products on protein functions have been documented (e.g. decreased solubility, aggregation, nutritional value). In bioactive peptides, however, the functional consequences of oxidative modification remain largely unexplored. Given their growing use in functional foods and nutraceuticals, understanding how ROS-induced structural changes alter peptide bioactivity, safety, and biological signalling remains a critical and currently underdeveloped area of research.

### Reactions of antioxidant peptides with secondary lipid oxidation products

Bioactive antioxidant peptides react with primary lipid oxidation products (e.g. lipid hydroperoxides) to form compounds similar to those in the preceding section. Reactions of the active peptides with secondary lipid oxidation products such as malondialdehyde (MDA), 4-hydroxy-2-nonenal (HNE), 4-oxo-nonenal, and truncated oxidized phospholipid fragments will also reshape peptide structures, stabilities, and biological functions. The exposure of peptides to oxidizing lipid environments will then lead to complex oxidative remodelling, often involving a mixture of radical-driven and nucleophile-electrophile mechanisms.

Reactive aldehydes modification of bioactive peptides will occur through Schiff base formation and Michael addition. MDA readily forms Schiff bases with Lys (K), His (H), Arg (R), Gln (Q), and Asn (N), producing both reversible imines and stabilized cyclic adducts. In contrast, HNE, 4-oxo-nonenal, and related α,β-unsaturated aldehydes will predominantly form Michael-type adducts with Cys, His, and Lys residues, yielding pyrrolic or hemiacetal structures characteristic of advanced lipoxidation end-products [56, 57, 64]. Cyclic hemiacetals, pyrrole adducts, or cross-linked structures, depending on the aldehyde structure, can also be formed.

**Fig. 6:**
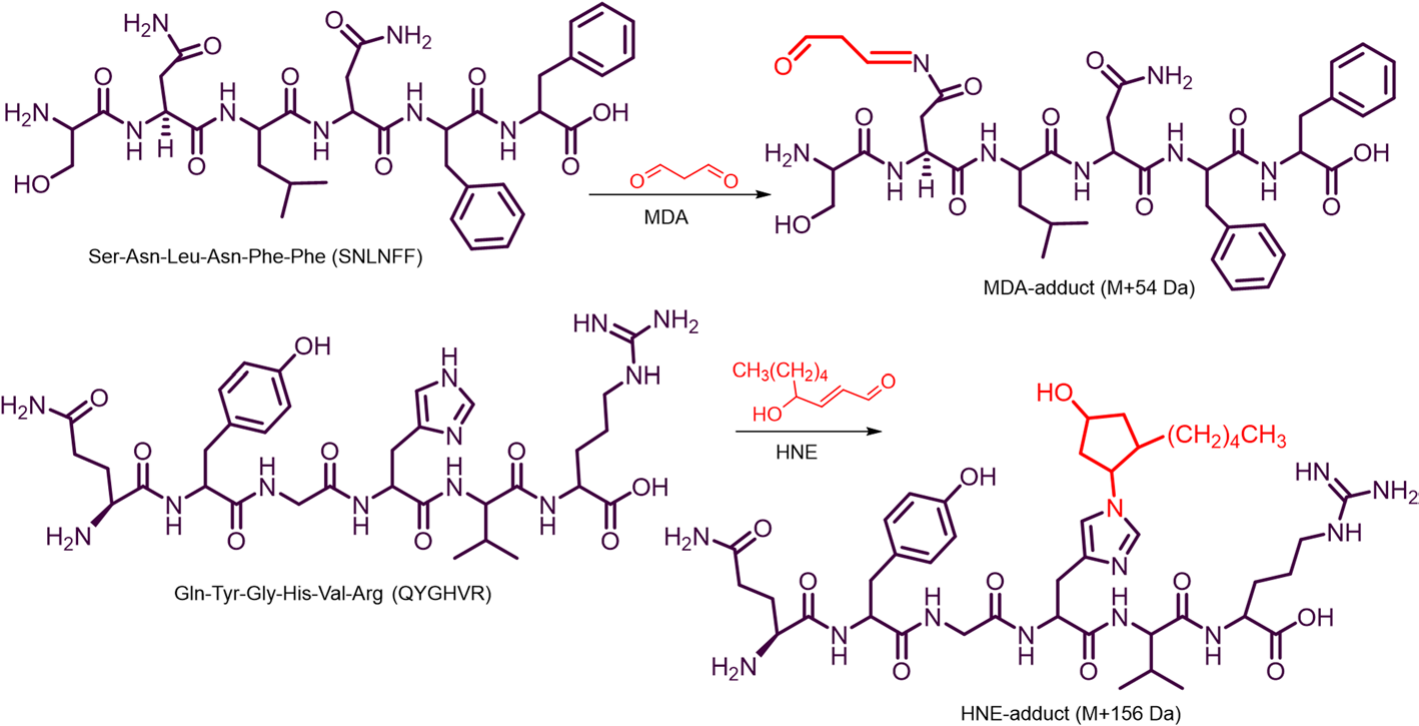
Examples of conjugated malondialdehyde and hydroxynonenal products of peptides SNLNFF and QYGHVR

Nucleophilic amines on antioxidant peptides from soy proteins reacted with MDA to form several Schiff base adducts characterized by + 54 Da, as well as the MDA-derived dihydropyridine (DHP)-lysine formed through a reaction with MDA and acetaldehyde, and characterized by a mass of + 134 Da [57]. Schiff base formation products were found on the side-chain groups of Lys, His, Arg, Gln, and Asn. In total, the researchers identified 53 peptides that reacted with MDA through Schiff base, with more than half taking place on the N-termini of the peptides. The antioxidant soy peptides also reacted with HNE to form both Schiff (+ 138 Da) and Michael adduct (+ 156 Da) products. Antioxidant peptides containing lysine also formed the pyrrole-type HNE adducts. Products derived from Michael addition were the primary ones for basic amines, except for lysine [57]. The structures of some of the soy antioxidant peptides and their adducts are illustrated in Fig. 6. A study on peptides ALPMHIR, LIVTQTMK, and VLVLDTDYK from β-lactoglobulin also identified MDA, HNE products [11] formed in a similar mechanism to those from soy.

Secondary lipid-oxidation adducts can markedly alter the physicochemical and biological properties of antioxidant peptides, raising safety concerns. Literature works on proteins have shown that modification with MDA can promote aggregation, reduce proteolytic digestibility, and allow structurally altered fragments to reach the intestine, where they may impair barrier integrity and contribute to hepatic dysfunction [65]. Other aldehyde-derived adducts, such as DHP-lysine, exhibit photoreactivity and cytotoxicity to human skin fibroblasts and keratinocytes due to the production of ROS [66]. MDA/HNE-derived pyrroles, which contain planar aromatic headgroups, can intercalate into membranes and disrupt their organization. Despite these documented effects of protein-bound MDA and HNE, the safety of antioxidant peptides modified by such aldehydes remains unexplored. Although one of the mechanisms of antioxidant peptides is to limit lipid hydroperoxide formation, and thus the generation of MDA and HNE, their aldehyde-conjugated products may still occur at low levels. Determining the concentrations of these modified peptides and assessing their functional and unintended activities remains essential.

### Reactions of antioxidant peptides with sugars

The reaction of antioxidant peptides with reducing sugars, commonly referred to as glycation, has traditionally been explored as a strategy to enhance peptide functionality, radical scavenging, and metal chelating properties [12, 67, 68]. In the early phase of glycation, nucleophilic amino groups on the antioxidant peptide side chain or N-terminus react with reducing sugar carbonyls to form Schiff base products, which undergo Amadori rearrangement to yield glucosamine-type intermediates. Continued heating promotes dehydration, cyclization, and fragmentation reactions that generate hydroxymethylfurfural, aldehydes, pyrazines, and other Maillard reaction-derived products [69, 70]. Many of these intermediates possess strong reducing power or contribute favourable sensory attributes, which has encouraged the application of glycation reactions in enhancing the antioxidant activities of peptides. The same reaction pathways can, however, progress further under thermal processing to yield heterocyclic compounds, including heterocyclic aromatic amines (HAAs) and other advanced glycoxidation products that carry recognized toxicological concerns [71]. When antioxidant peptides are deliberately glycated to enhance functionality, it then becomes critical to examine whether such processing unintentionally increases HAA formation, especially under conditions involving high temperature, prolonged heating, or low moisture. Quantifying HAAs and related glycoxidation end-products should therefore be a standard component of safety assessment for glycated peptide ingredients.

Animal and cell-based studies further underscore the need for caution. For example, glucose-glycated silver carp peptides, despite exhibiting improved in vitro antioxidant activity, were shown to increase oxidative stress in diabetic mice, suggesting that in vivo behaviour can diverge markedly from in vitro antioxidant readouts [72]. Additionally, glycated peptide consumption may also influence metabolic outcomes, as glucose glycated anchovy peptides caused increased body weight in male mice while decreasing weight in female mice, indicating possible sex-dependent metabolic effects [73]. At the cellular level, galactose glycated gelatin peptides inhibited Caco-2 cell proliferation (IC_50_ 1.7 mg/mL), whereas glycation with whey peptides enhanced proliferation by approximately 30% at 1 mg/mL [74, 75]. These variable outcomes highlight that glycation does not uniformly improve bioactivity and may instead impart biological activities unrelated or even counteractive to the intended antioxidant function.

Antioxidant peptides are valued for their ability to quench radicals and minimize the oxidation of food and biological molecules. In the process, they may form secondary products such as peptide hydroperoxides, carbonyls, glycated compounds, and lipoxidation adducts, which can potentially enhance certain functional properties of the antioxidant peptides. However, these peptide-mediated reactions can also introduce structural and toxicological uncertainties. As a result, the evaluation of bioactive peptides should extend beyond their antioxidant capacity to include assessments of cellular responses and metabolic effects related to undesirable reaction products.

Arylamide is a Maillard reaction product in heat-treated foods that is listed as probably carcinogenic to humans; meanwhile, human exposure (0.02–6.41 µg/kg bodyweight/day) is well below oral toxicity in animal models (LD50– 195 mg/kg, mice; 150–413 mg/kg, rats) [76, 77]. The vast majority of dietary acrylamide is formed between the free amino acid asparagine and reducing sugars [76]. The contribution of bioactive peptides can therefore be considered minimal.

### Formation of lysinoalanine

In addition to oxidative and glycative modifications, lysinoalanine represents a processing-induced hazard. It is formed under alkaline and thermal conditions, where the lysine side chain reacts with dehydroalanine derived from serine, cysteine, or cystine residues [78]. Lysinoalanine has been detected in rice, soy, and egg white proteins at concentrations ranging approximately from 0.3 to 25 g/kg protein, depending on alkali strength, heating temperature, and treatment duration, with higher levels observed under more severe processing conditions [78–80]. The hydrolysis of these alkaline-treated proteins can therefore yield protein hydrolysates containing lysinoalanine. Lysinoalanine can also occur when hydrolyzed proteins are subsequently heat-treated.

In its free form, lysinoalanine exerts adverse biological effects, as it can form chelates with metalloenzymes, which may induce nephrocalcinosis and cause the formation of giant renal cells and necrosis of renal tubular cells [78]. This is illustrated, for example, by a study in which feeding synthesized lysinoalanine to rats at dietary levels of 0.01% and above induced typical renal nephrocytomegalia, whereas no toxicity was observed when administered in the form of alkali-treated casein or soy proteins containing 0.6% lysinoalanine [81]. In another study, diets containing 20% alkali-treated soy protein (about 2630 ppm lysinoalanine) produced nephrocytomegalia in male rats, while no toxicity was observed in diets containing alkali-treated lactalbumin at equivalent lysinoalanine content [82]. These data demonstrate that lysinoalanine toxicity depends on its chemical form and the protein to which it is linked. Bioactive peptides are shorter in sequence, and the effects of their lysinoalanine derivatives may differ from those of lysinoalanine in intact proteins. The toxicological evaluation of lysinoalanine-containing peptides, however, is still lacking.

### Potential role of gut microbiota in the transformation of peptides and modified peptides

Native and oxidatively modified peptides that escape host digestion will enter the large intestine, where they can further be hydrolyzed and fermented by microbial enzymes into shorter sequences and a diverse range of secondary metabolites. Native peptides are capable of metabolizing into beneficial compounds such as short-chain fatty acids (SCFAs) and reactive sulfur species, which contribute to redox homeostasis by enhancing antioxidant enzyme activity, maintaining intestinal barrier integrity, and modulating immune responses, as summarized in recent review publications [83, 84]. In contrast, oxidatively modified bioactive peptides may reduce degradability, redirect metabolic pathways, or lead to the persistence of modified peptides within the gut environment, ultimately affecting their contribution to redox processes.

Microbial metabolism of peptides can generate both beneficial and potentially harmful metabolites depending on the substrate structures. In vitro colonic fermentation showed that hydrolyzed soy proteins increased SCFAs (acetate, lactate, propionate, butyrate) while increasing Lactobacilli and Clostridia species, with a concomitant reduction in Bifidobacterium and Bacteroides [84]. Oxidatively modified peptides are likely more prone to generating reactive or pro‑oxidant intermediates due to their inherent electrophilic functionalities, which can contribute to dysbiosis, increased intestinal permeability, and enhanced inflammatory signalling, as oxidative stress and gut dysbiosis are interconnected [83, 85].

### Site specific identification of oxidized peptides using peptidomics

Recent advances in mass spectrometry-based proteomics and peptidomics have enabled the emergence of oxidative peptidomics, which allows site-specific identification of oxidative modifications in proteins and derived peptides [86–88]. For intact proteins, analytical workflows typically involve reduction and alkylation of cysteine residues prior to enzymatic digestion, ensuring stabilization of disulfide bonds and improved peptide recovery for subsequent LC–MS/MS analysis. In contrast, protein hydrolysates, which already consist of short peptides do not require further digestion, and can be analyzed directly [86, 88]. During data processing, database searches incorporate a broad range of variable oxidative modifications, including mono-oxidation (+ 16 Da), di-oxidation (+ 32 Da), and tri-oxidation (+ 48 Da) on residues such as cysteine, methionine, tyrosine, histidine, tryptophan, and phenylalanine, as well as specific transformations such as kynurenine (+ 4 Da), hydroxykynurenine (+ 20 Da), trihydroxyphenylalanine quinone (+ 30 Da), and lipid-derived adducts including malondialdehyde (MDA, + 54 Da) and 4-hydroxy-2-nonenal (HNE, + 156 Da) [86, 88]. Localization of these modifications is achieved through tandem MS/MS fragmentation, where b- and y-ion series enable precise mapping of mass shifts along the peptide backbone, allowing differentiation of oxidation occurring on specific residues (e.g., cysteine versus tryptophan or tyrosine) even when identical nominal mass increases are present. These approaches have been applied to oxidized whey protein systems where heat-induced oxidation targets cysteine, methionine, and tryptophan and promotes disulfide rearrangement and aggregation [86]. Similarly, LC–MS/MS-based peptidomics has been used to characterize lipoxidation in fish myofibrillar proteins, revealing extensive site-specific modifications induced by oxidized linoleic acid and 4-hydroxy-2-nonenal on lysine, histidine, and aromatic residues [87, 89]. The approach has also been extended to peptide systems, such as oxidized fish-skin collagen hydrolysates, where oxidative modifications (e.g., oxidation, di-oxidation, carbonylation, and MDA/HNE adducts) were mapped and shown to correlate with reduced antioxidant activities [88]. Despite these advances, the application of oxidative peptidomics specifically to food-derived bioactive peptides remains limited, highlighting an important opportunity to systematically map peptide oxidation products and link structural changes to antioxidant function and safety outcomes.

## Toxicity and safety assessment of antioxidant peptides

The evaluation of the toxicity and safety of antioxidant peptides intended for use in foods, functional ingredients, or therapeutic preparations is essential. Although many of these peptides originate from edible proteins and thus appear inherently safe, their extraction, concentration, enzymatic release, further hydrolysis, or formulation may produce peptides with biological activities distinct from their parent proteins [90, 91]. Food peptides exhibit a wide range of cellular and systemic effects, including beneficial antioxidant actions, potentially unintended cytotoxic, genotoxic, pro-apoptotic, membrane-lytic, immunomodulatory, or organ-specific toxicities under certain conditions (e.g., high concentrations, structural changes, or susceptible cell types). Safety assessment is therefore crucial to determine appropriate dosing, identify sequence-specific hazards or hazards associated with modified peptides as they are chemically more reactive than their parent proteins.

### Overview of toxicity assessment methods

Toxicity evaluation often starts with in vitro assays, which can progress to ex vivo or alternative organism models, and eventually to animal studies with each type of analysis capturing different aspects of biological risk. Initial cellular toxicity assays evaluate membrane integrity (lactate dehydrogenase (LDH) release), mitochondrial activity (MTT), lysosomal function (Neutral Red Uptake), apoptosis/necrosis (Annexin-V/PI), and mitochondrial health (JC-1 dye and tetramethylrhodamine, methyl ester dye (JC-1/TMRM), and adenosine triphosphate measurements) [92, 93]. Genotoxicity is assessed through the comet assay and micronucleus test [92]. High-content imaging enables simultaneous visualization of ROS accumulation, DNA injury, and cell structural changes. For intestinal-targeted peptides, barrier integrity is monitored using transepithelial electrical resistance (TEER) in Caco-2 monolayers. Animal studies add essential insights into systemic exposure, organ-specific toxicity, and long-term effects such as hepatotoxicity, nephrotoxicity, neurotoxicity, reproductive toxicity, and developmental outcomes. Standard endpoints include lethal dose determination, biochemical markers such as alanine aminotransferase, aspartate aminotransferase, creatinine, histopathology, micronucleus and comet genotoxicity assays, behavioural or cognitive testing (for neurotoxic peptides), and lifespan or stress-resistance evaluations in alternative organisms like zebrafish and *C. elegans* [94–96]. Together, these tools provide a multidimensional safety profile necessary for evaluating antioxidant peptides before incorporation into food or therapeutic systems.

### Cellular toxicity of antioxidant peptides

Despite the expanding interest in antioxidant peptides and their therapeutic or food-related applications, systematic evaluation of their cellular toxicity remains limited. In most studies, cytotoxicity assays are indeed performed, but they are typically used only to confirm that the peptide concentration selected for bioactivity testing (antioxidant, anti-inflammatory, antihypertensive) does not adversely affect cell viability. As a result, most publications report toxicity outcomes incidentally rather than as a primary objective, and only a fraction provide detailed IC_50_ values or mechanistic analyses. This has created a gap in the toxicological understanding of antioxidant peptides, especially for concentrations above those used for bioactivity assays.

A major complication in interpreting peptide toxicity arises from the use of both normal and tumour cell lines. Normal cell models that have been used include MRC-5 (human lung fibroblasts), HEK-293 (embryonic kidney, though partly transformed), human red blood cells, Vero cells (African green monkey kidney), and Caco-2 (non-tumor intestinal epithelium, although possessing tumour origin, they differentiate into normal enterocyte-like monolayers). These lines reflect more physiologically relevant toxicity, including membrane integrity (LDH release), hemolysis, and TEER barrier disruption. Tumour cells such as A549, H1975, HepG2, HCT-116, and MCF-7 often retain normal cellular features, such as epithelial polarity, metabolic enzymes, and membrane transporters, making them partially representative of normal tissues while still exhibiting tumor-specific vulnerabilities; as such, they are often used for metabolic and toxicological evaluation. Specifically, for example, Caco‑2 cells grown to confluence spontaneously differentiate into enterocyte‑like cells of the small intestine, while HepG2 cells maintain liver‑specific metabolic functions such as producing albumin, transferrin, and coagulation factors, and MCF7 cells retain estrogen receptors and remain responsive to estrogen [97–99].

Across these models, several antioxidant or food-derived peptides demonstrated distinct, dose-dependent cytotoxicity profiles (Table 3). Piscidin-4 from tilapia reduced cell viability by 30–35% in lung cancer lines (H1975 and A549) and by 15% in normal MRC-5 fibroblasts at 15 µg/mL [100], while the milk-derived peptide LRLKKYKVPQL caused 23.54% hemolysis in human red blood cells at 512 µM [101]. Multiple wheat-germ peptides (KELPPSDADW, SSDEEVREEKELDLSSNE, SGGSYADELVSTAK, MDATALHYENQK, TVGGAPAGRIVME, and VGGIDEVIAK) exhibited cytotoxic effects in A549 cells, with IC_50_ values ranging from 2.3 to 11.2 µM [102]. These data indicate that peptide‑mediated cytotoxicity can extend to non-tumour cells at comparable concentrations, that cationic peptides may possess hemolytic activity, and that acidic or mixed‑polarity motifs can differentially compromise cell viability. Other examples showed that protein hydrolysates from hyacinth bean triggered apoptosis in A549, MCF-7, and HEK-293 cells, as shown by Annexin V/PI staining and caspase-3/7 activation, with MTT-derived IC_50_ values between 9.80 and 119.6 µg/mL depending on the cell line [103]. Soy-derived peptides IYVVDLR and IYVFVR similarly could reduced Caco-2 viability at concentrations above 100 µM [104]. Gluten peptides elicited inflammatory, genotoxic, cytotoxic, and epithelial stress responses in Caco-2 intestinal models starting at 500 µg/mL, consistent with their established roles in celiac-associated pathology. Antioxidant peptides overall have shown little cellular toxicity near their effective antioxidant doses, meanwhile the limited availability of detailed cytotoxicity data, combined with examples of membrane damage, apoptosis, genotoxicity, and immune activation, underscores the need for standardized and thorough evaluation.

**Table 3:** Peptides and their effects in cellular models

Peptide / Hydrolysate	Source	Cell model	Assay(s)	Dose / effects
Piscidin-4 (FIHHIIGGLFSAGKAIHRLIRRRRR)	Tilapia	H1975	Trypan blue	15 µg/mL → 30% decrease in viable cells (cytotoxic) [100]
Piscidin-4	Tilapia	A549	Trypan blue	15 µg/mL → 35% decrease in viable cells [100]
Piscidin-4	Tilapia	MRC-5	Trypan blue	15 µg/mL → 15% cytotoxicity in normal fibroblasts [100]
LRLKKYKVPQL	Milk	Human red blood cells	Hemolysis	512 µM → 23.54% hemolysis (membrane damage) [101]
Pepsin-pancreatin digest	Amaranth	Caco-2 TC7	LDH	Increased LDH release → indicates membrane lysis/cytotoxicity [93]
KELPPSDADW	Wheat germ	A549	Trypan blue	IC_50_ = 2.34 µM → cytotoxicity [102]
SSDEEVREEKELDLSSNE	Wheat germ	A549	Trypan blue	IC_50_ = 2.34 µM → cytotoxic [102]
SGGSYADELVSTAK	Wheat germ	A549	Trypan blue	IC_50_ = 7.2 µM → cytotoxicity [102]
MDATALHYENQK	Wheat germ	A549	Trypan blue	IC_50_ = 10.7 µM → cytotoxic [102]
TVGGAPAGRIVME	Wheat germ	A549	Trypan blue	IC_50_ = 9.7 µM → cytotoxicity [102]
VGGIDEVIAK	Wheat germ	A549	Trypan blue	IC_50_ = 11.2 µM → cytotoxicity [102]
Whey protein hydrolysate (WPH)	Milk	Vero C-76	Neutral Red Uptake	IC_50_ = 5.7 mg/mL → cytotoxicity [92]
WPH < 3 kDa	Milk	Vero C-76	NRU	IC_50_ = 2.3 mg/mL → cytotoxicity [92]
Hyacinth bean pepsin hydrolysate	Hyacinth bean	A549	Trypan blue; Annexin-V/PI; Caspase-3/7	IC_50_ = 119.6 µg/mL (cytotoxic); Increased apoptosis and caspase-3/7 [103]
Hyacinth bean pepsin hydrolysate	Hyacinth bean	MCF-7	Trypan blue; Annexin-V/PI; Caspase-3/7	IC_50_ = 9.80 µg/mL (cytotoxic); increased apoptosis and caspase [103]
Hyacinth bean pepsin hydrolysate	Hyacinth bean	HEK-293	Trypan blue; Annexin-V/PI; Caspase-3/7	IC_50_ = 13.86 µg/mL (cytotoxc); apoptosis and caspase-3/7 activation [103]
Trypsin hydrolysate	Amaranth	MCF-7	MTT; Annexin-V	IC_50_ = 3.87 µg/mL (cytotoxicity); Increased apoptosis [105]
Trypsin hydrolysate	Amaranth	A549	MTT; Annexin-V	IC_50_ = 14.10 µg/mL (cytotoxic); increased caspase-3/7 [105]
Pepsin-treated casein fraction	Goat milk	HCT-116	MTT; Annexin-V/PI	20% decrease in viable cells (cytotoxic); apoptotic cell death [106]
IYVVDLR / IYVFVR	Soy =	Caco-2	Tetrazolium salts (CCK-8)	Cytotoxic above 100 µM [104]
Gliadin hydrolysates and α-2 gliadin 33-mer peptide	Wheat/gluten	Caco-2	MTT, inflammatory markers	33-mer (250 ug/mL); hydrolysate (1 mg/mL); upregulated nitric oxide (NO), cytokine (TNF-α and IL-1β) [107]
Melittin peptides	Bees	RAW264.7, HEK293T, & HMEC-1	WST-8	25 µg/mL → >90% reducing of cell viability, induced Ca^2+^ signaling [108]
DGIFVLNY; IPTDEK	*Zingiber cassumunar*	Caco-2	MTT; Annexin-V/PI; Caspase-3/9	5 µM → 30–40% reduced viability; Increased apoptosis, caspase-3/9 [109]

### Toxicity and safety in animal models

Animal models are indispensable for evaluating the systemic toxicity of antioxidant peptides because they capture whole-organism processes, which might include absorption, circulation, metabolism, and excretion, as well as developmental, neurobehavioral, and organ-specific toxicities that cannot be inferred from cell-based assays. Despite this importance, in vivo studies on antioxidant peptides are far fewer than cellular investigations. This could be because producing the large quantities of highly purified peptide required for animal dosing is costly. Consequently, the limited number of existing in vivo studies is mostly on protein hydrolysates or peptide-enriched fractions. For food applications, this approach is often still appropriate, since hydrolyzed proteins and their fractions are more cost-effective to produce.

Among the few available investigations (Table 4), whey protein hydrolysates and their < 3 kDa fractions demonstrated important toxicity considerations. In mice, they caused genotoxic effects at elevated doses, with micronucleus assays showing increased micronucleated polychromatic erythrocytes at 200 mg/kg body weight and comet assays detected DNA strand breaks at 50–100 mg/kg [92]. These findings do not contradict the antioxidant or health-promoting actions attributed to peptides or protein hydrolysates, but they highlight that at certain concentrations, protein hydrolysates may generate reactive metabolites or interact unfavourably with nucleic acids. A group of well‑characterized toxic peptides comprises the cyclic compounds α‑amanitin, β‑amanitin, phallacidin, and phalloidin from certain mushroom species [110]. These peptides induce rapid, dose‑dependent liver and kidney failure, reflected by elevated alanine aminotransferase (ALT) and aspartate aminotransferase (AST), urea nitrogen, and creatinine, alongside histopathological evidence of steatosis, necrosis, and mitochondrial collapse [110]. Together, these findings illustrate that peptide toxicity can be highly organ‑specific and sequence‑dependent.

Complementary insights arise from non-mammalian vertebrate systems, particularly the zebrafish (Danio rerio), which is appropriate for early activity or toxicity screening. Several antioxidant peptides derived from egg-lysozyme (VAWRNRCKGTD, WRNRCKGTD, AWIRGCRL, WIRGCRL, and IRGCRL) were non-toxic to embryos and larvae at concentrations below 50 µg/mL, but toxicity manifested at higher doses [111]. For example, AWIRGCRL produced 50% embryo mortality at 800 µg/mL, despite displaying the strongest ABTS scavenging activity [111]. This illustrates that antioxidant potency does not preclude toxicity, particularly when concentrations exceed physiological or food-relevant levels. Similarly, protein hydrolysates from the red seaweed *Palmaria palmata* revealed a dose-response gradient in zebrafish. Larvae exposed to 1 mg/mL showed normal morphology, while those exposed to 5 mg/mL exhibited developmental abnormalities such as swollen yolk sacs and curved spines [112]. Walnut protein hydrolysates showed a maximum tolerated concentration of 500 µg/mL, and the isolated walnut-derived peptide IRALPEEVL was tolerated up to 1000 µg/mL [113]. At safer doses, IRALPEEVL penetrated the blood–brain barrier via caveolin-1 and ameliorated cognitive impairments induced by bisphenol AF [113], demonstrating that some antioxidant peptides can exert neuroprotective benefits when administered within their non-toxic range. In contrast, antioxidant peptides derived from phycobiliprotein (MHLWAAK, MAQAAEYYR, and MDYYFEER) displayed embryotoxicity above 160 µg/mL [55], indicating narrower safety margins for specific peptide structures.

**Table 4:** Peptides and their effects in animal models. ALT: alanine aminotransferase; AST: aspartate aminotransferase;, BUN: blood urea nitrogen

Peptide / Compound	Source	Animal	Assay/Readout	Dose	Key Effects
Whey protein hydrolysate (WPH)	Milk	Mouse	Micronucleus assay (bone marrow)	200 mg/kg bw	Increased micronucleated polychromatic erythrocytes → genotoxicity [92]
WPH	Milk	Mouse	Comet assay (DNA damage)	≥ 100 mg/kg bw	DNA strand breaks detectable at 100 mg/kg [92]
WPH (< 3 kDa)	Milk	Mouse	Comet assay	≥ 50 mg/kg bw	DNA damage detectable at ≥ 50 mg/kg [92]
Cyclic mushroom peptides	*Amanita* spp.	Mouse	ALT, AST, BUN, creatinine; histopathology	Dose-dependent	Hepato- & nephrotoxicity; steatosis, necrosis, mitochondrial collapse; fatal at high dose [110]
VAWRNRCKGTD, WRNRCKGTD, AWIRGCRL, WIRGCRL, & IRGCRL	Egg white	Zebrafish embryos & larvae	Embryotoxicity	≤ 50 µg/mL safe; >50 µg/mL toxic	Safe at low doses; Mortality increases above 50 µg/mL [111]
AWIRGCRL,	Egg white	Zebrafish embryos & larvae	Embryotoxicity	800 µg/mL	50% mortality despite good ABTS antioxidant activity [111]
Palmaria palmata hydrolysate	Red seaweed	Zebrafish larvae	Morphological toxicity	1 mg/mL safe; 5 mg/mL toxic	5 mg/mL → swollen yolk sac, curved spine [112]
Walnut protein hydrolysate	Walnut	Zebrafish	Toxicity threshold	Max tolerated = 500 µg/mL	Above threshold → embryotoxicity [113]
IRALPEEVL (walnut peptide)	Walnut	Zebrafish	Cognitive and developmental assessment	Max tolerated (1000 µg/mL)	Safe up to 1000 µg/mL; crosses BBB via caveolin-1; improves bisphenol-AF-induced cognitive deficits [113]
MHLWAAK, MAQAAEYYR& MDYYFEER	Phycobiliprotein	Zebrafish embryos	Embryotoxicity	> 160 µg/mL	Embryotoxicity at higher doses [55]

### Limitations of safety assessment

A limitation in evaluating the safety of food-derived antioxidant peptides and bioactive peptides in general is the difficulty in estimating their actual dietary exposure under normal consumption conditions, as purified peptides are not yet widely incorporated into foods at defined levels. However, available in vivo studies on protein hydrolysates and peptides, summarized in recent review articles, provide a framework to estimate exposure [114–116]. Human intervention studies have assessed doses of 1–50 mg/day (0.014–0.71 mg/kg bw/day) for purified peptides, whereas peptide-rich protein hydrolysates derived from casein, whey, soy, pea, egg, and fish proteins have been investigated at 0.2–150 g/day (0.003–2.14 g/kg bw/day), with most studies using less than 35 g/day (0.5 g/kg bw/day) [114]. Peptides (VPP, ad IPP) from milk proteins alone or in combination with casein hydrolysates, whey protein hydrolysates, and other hydrolysates, which have been evaluated in human studies delivered as beverages, tablets, or functional food matrices and have demonstrated good tolerability under both acute and chronic intake conditions [114]. At 3–50 mg/day, VPP and IPP show no adverse effects, while gram-scale intake of hydrolysates across multiple food sources has not resulted in toxicity [114, 116]. In rats, bovine hair hydrolysates exhibit no acute toxicity, mutagenicity, or genotoxicity, following acute oral gavage at a dose of 10 g/kg body weight [115]. Collectively, evidence from recent reviews suggests that the probability of toxicity is low for bioactive peptides. Nevertheless, higher exposure scenarios such as concentrated supplements or oxidatively modified peptide products may require further investigation through dose-response analysis and long-term safety assessment.

## Perspectives and conclusion

Many antioxidant peptides have demonstrated protective effects in cellular and animal models; however, the overall evidence supporting their safety in humans remains limited beyond the assumption of safety derived from their origin as food proteins. Most available toxicological data rely on in vitro assays and short-term animal studies, which do not fully capture long-term exposure, cumulative metabolic effects, or interactions with complex biological systems such as the human microbiome, immune system, and food matrix. These limitations highlight the need to move beyond generalized safety assumptions and toward evidence-based evaluation of individual peptides and their modified forms.

A main factor influencing both efficacy and safety is digestive stability and bioavailability. Many antioxidant peptides are susceptible to gastrointestinal proteolysis, and only small peptides (primarily di- and tri-peptides) have been reported to absorb via peptide transporter systems such as PepT1 [117, 118]. Larger peptides may instead exert local effects within the gastrointestinal tract or be further metabolized by gut microbiota into smaller bioactive or inactive fragments [119]. Importantly, oxidative, glycated, and lipoxidized modifications can alter peptide stability, enzymatic resistance, absorption, and metabolic fate, potentially reduce bioactivity or generate metabolites with distinct redox or toxicological properties. Therefore, safety assessment should incorporate digestive models, intestinal transport studies, and microbiota-mediated metabolism to better predict the biological fate of both native and modified peptides. Modified peptides may exhibit altered bioactivity, increased reactivity, or potential toxicity. In addition, peptides derived from allergenic proteins may retain or acquire immunogenic properties, emphasizing the need for sequence-specific and source-specific safety evaluation, including allergenicity prediction and validation.

Given these complexities, establishing the safety of antioxidant peptides requires a multitiered and integrative evaluation framework, including: (i) in silico screening (toxicity prediction, allergenicity, and structure-toxicity relationships), (ii) in vitro assays (cytotoxicity, oxidative stress response, intestinal barrier integrity, and mitochondrial function), (iii) digestion and bioavailability assessment (peptide transport models, metabolite identification), (iv) microbiota interaction studies (biotransformation and redox modulation), and (v) in vivo validation (subchronic toxicity, organ-specific effects, and exposure-response relationships).

To advance the field, several research directions are needed to strengthen and refine current safety evaluation approaches. They include:

- Systematic mapping of oxidative peptide modifications using high-resolution LC–MS/MS–based oxidative peptidomics to identify previously uncharacterized products.
- Development of quantitative structure-toxicity relationship (STR) databases linking specific oxidative modifications to biological outcomes.
- Integration of AI-driven predictive models to improve the reliability of toxicity, bioavailability, and metabolism predictions.
- Improved quantification of human exposure levels and dose-response relationships, including chronic and high-dose intake scenarios.
- Deeper mechanistic understanding of microbiota-mediated transformations of oxidized peptides, particularly their role in dysbiosis and oxidative stress.
- Development of standardized and harmonized regulatory frameworks tailored to food, nutraceutical, and pharmaceutical applications.

Even peptides regarded as beneficial at low concentrations may exhibit different biological effects at higher doses or following structural modification. Accordingly, antioxidant peptides should not be viewed as universally safe molecules but rather as chemically dynamic entities whose safety depends on structure, dose, transformation, and biological context. Integrating mechanistic, analytical, and toxicological approaches will be essential to fully harness their health benefits while minimizing unintended risks and ensuring safe application in food and health-related systems.

## Data Availability

No datasets were generated or analysed during the current study.
